# Neuronal Nitric Oxide Synthase Knockdown Within Basolateral Amygdala Induces Autistic-Related Phenotypes and Decreases Excitatory Synaptic Transmission in Mice

**DOI:** 10.3389/fnins.2020.00886

**Published:** 2020-08-31

**Authors:** Xiaona Wang, Chao Gao, Yaodong Zhang, Jinxiu Xu, Quanfeng Fang, Lingshan Gou, Zhigang Yang, Daoqi Mei, Leiming Liu, Linfei Li, Jing Liu, Huichun Zhang, Yinsen Song

**Affiliations:** ^1^Henan Key Laboratory of Children’s Genetics and Metabolic Diseases, Henan Neurodevelopment Engineering Research Center for Children, Children’s Hospital Affiliated to Zhengzhou University, Zhengzhou, China; ^2^Department of Rehabilitation, Children’s Hospital Affiliated to Zhengzhou University, Zhengzhou, China; ^3^School of Basic Medicine, Sanquan Medical College, Xinxiang, China; ^4^Healthcare Department, Children’s Hospital Affiliated to Zhengzhou University, Zhengzhou, China; ^5^Center for Genetic Medicine, Xuzhou Maternity and Child Health Care Hospital, Xuzhou, China; ^6^Department of Neurology, Children’s Hospital Affiliated to Zhengzhou University, Zhengzhou, China; ^7^Department of Medical Assistance, Children’s Hospital Affiliated to Zhengzhou University, Zhengzhou, China; ^8^People’s Hospital Affiliated to Henan University of Chinese Medicine, Zhengzhou, China

**Keywords:** neuronal nitric oxide synthase, autism spectrum disorder, interneuron, synaptic transmission, basolateral amygdala

## Abstract

Autism spectrum disorder (ASD) is a heterogeneous group of neurodevelopmental disorders characterized by deficits in communication, impaired social interaction, and repetitive or restricted interests and behaviors. We have recently shown that neuronal nitric oxide synthase (nNOS) expression was reduced in the basolateral amygdala of mice after postnatal valproic acid exposure. However, the specific role of nNOS downregulation in mice remains to be elucidated. Herein, we investigated the behavioral alternations of naive mice with a recombinant adeno-associated virus (rAAV)-mediated knockdown of nNOS in a comprehensive test battery, including the social interaction, marble burying, self-grooming, and open field tests. Further, the electrophysiological and surface expression changes induced by nNOS deficiency of the basolateral amygdala in these animals were examined. Our results show that nNOS knockdown displayed typical symptoms of ASD-like behaviors, such as reduced social interaction and communication, elevated stereotypes, and anxiety in mice. Surprisingly, we found that nNOS knockdown exhibited greatly reduced excitatory synaptic transmission concomitant with the lower surface expression of GluN2B-containing N-methyl-D-aspartate receptors and postsynaptic density protein 95 in mice. These findings support a notion that dysregulation of nNOS might contribute to ASD-associated phenotypes, with disease pathogenesis most likely resulting from deficits in excitatory synaptic transmission.

## Introduction

Autism spectrum disorder (ASD) is a highly disabling neurodevelopmental disorder defined by a triad of core symptoms, including impairments in social interactions, restricted interests, stereotypes/restricts, and anxiety-like behavior (Lin et al., 2013). Several brain structures and functions have been proposed to underlie multiple abnormal behaviors related to ASD, including the basolateral amygdala (BLA). Studying in animals has elegantly demonstrated that GABAergic interneurons within the BLA are strongly involved in ASD-related phenotypes (Prager et al., 2016). Indeed, our work shows that inhibitory synaptic transmission in interneurons was decreased in the BLA of valproic acid (VPA)-treated animals (Wang et al., 2018b). Collectively, these results confirm that excitatory/inhibitory synaptic dysfunction that results in a hypersynchronous discharge of neurons may contribute to the manifestation of ASD-associated symptoms (Todd and Anderson, 2009; Li et al., 2018).

Increasing evidence suggests that glutamate receptors associated with the development of autistic symptoms (Tarabeux et al., 2011; Lee et al., 2015a; Kim et al., 2019). In particular, it has been demonstrated that constitutive N-methyl-D-aspartate (NMDA) receptors and α-amino-3-hydroxy-5-methyl-4-isoxazolepropionic acid (AMPA) receptor hypofunction in mice were associated with deficits in social and communicative functioning as well as elevated repetitive behaviors (Gandal et al., 2012; Irie et al., 2012). NMDA receptors can enhance excitatory synaptic transmission induced by prenatal exposure to VPA (Lee et al., 2017). GLuN2B subunits of NMDA receptors are mainly expressed in the interneurons and mediate excitatory synaptic transmission in the amygdala (Nörenberg et al., 2012; Bacq et al., 2018). Of note, studies have shown that neuronal nitric oxide synthase (nNOS) binds to a scaffolding protein, postsynaptic density protein 95 (PSD-95) (Colciaghi et al., 2019). Recruitment of PSD-95 and other PSD-95-interacting membrane molecules to the synapse implicates in trafficking and clustering of NMDA receptors and AMPA receptors, consequently affecting synaptic function (Morimura et al., 2017). Nevertheless, it is presently undetermined that excitatory neurotransmitter and expression of ionotropic glutamate receptor subunits in nNOS interneurons trigged by nNOS deficiency are affected in native animals.

nNOS-containing interneurons are predominantly expressed in the cortex, hippocampus, and BLA in rodents (Tricoire et al., 2010; Wang et al., 2017). Multiple lines of evidence indicate that nNOS-positive cells are implicated in the regulation of synaptic transmission and plasticity (Armstrong et al., 2012; Lange et al., 2012). Specifically, Li and colleagues reported that the firing of hippocampal nNOS neurogliaform cells induces excitatory postsynaptic potential (Li et al., 2014). It was shown that nNOS deficiency impairs long-term memory of olfactory fear learning (Pavesi et al., 2013). nNOS knockout mice are reportedly hyperactive and display abnormal social, aggressive, and impulsive behaviors as well as cognitive impairments (Gao and Heldt, 2015). Noticeably, we have recently reported that downregulated nNOS of the BLA is a neuropathological finding in the VPA-treated mice (Wang et al., 2017, 2018a). Drawing on these findings, we, therefore, hypothesize that nNOS may regulate excitatory glutamatergic synaptic transmission concerning autistic behaviors.

In the current study, we first examined the behavioral alternations in recombinant adeno-associated virus (rAAV)-mediated knockdown of nNOS in native mice. Then, the changes in electrophysiological and surface expression in mice induced by nNOS deficiency within the BLA were studied. It is indicated that the down-expression of nNOS contributes to a spectrum of phenotypes associated with ASD. Moreover, we observed the dramatic decrease in excitatory synaptic transmission, consistent with reduced GLuN2B surface levels and PSD-95 in nNOS-deficient mice. Overall, our results demonstrated that nNOS knockdown leads to disturbance of BLA function and is liable for the evolvement of typical ASD-related phenotypes.

## Materials and Methods

### Animals

Animal experiments were approved by the Institutional Animal Ethics Committee of Shandong University, following the National Institutes of Health Guidelines for the Care and Use of Laboratory Animals. All measures were undertaken to minimize animal suffering and the number of animals used. Male C57BL/6 mice (20–24 g) were used for the study where rAAV vectors were infused into the BLA. All subjects were maintained in plastic **c**ages at a temperature of 22 ± 1°C in a clean environment under a 12:12-h light/dark cycle, with free access to food and water.

### Neuronal Nitric Oxide Synthase Down-Expression by Adeno-Associated Virus Vector Injection

Packages of rAAVs (rAAV2/9) were provided by Biopharmaceutical Technology Co., Ltd. (Shanghai, China). A U6 promoter was used to down express the nNOS gene (AAV-CMV-eGFP-U6-nNOS). Its ability to decrease nNOS expression in mice was verified in HEK293T cells. DNA transfection was performed using Lipofectamine 2000 (Invitrogen, Thermo Fisher Scientific, Inc.) according to the manufacturer’s recommendation. In brief, the day before transfection, cells reached 85% confluence. Then, the cells were transfected with the plasmid DNA and Lipofectamine 2000 at a ratio of 10 μg DNA:120-μl lipid per well in serum-free Opti-minimal essential medium (Gibco^TM^) at 37°C for 4 h. Subsequently, cells were grown for 48 h in Dulbecco’s modified Eagle medium containing fetal bovine serum. The control short hairpin RNA (shRNA) was a scrambled shRNA with an empty vector expressing green fluorescent protein (GFP) (rAAV-GFP). The scrambled shRNA sequence was 5′-TTCTCCGAACGTGTCACGT-3′. Viral titers were 4.57 × 10^12^ Tu/ml for rAAV-nNOS and 6.29 × 10^12^ Tu/ml for rAAV-GFP. Additionally, Supplementary Figure 1 shows an AAV vector spectrum graph. Supplementary Figure 2 demonstrates the recombinant AAV vector by DNA sequencing. Supplementary Figure 3 displays fluorescence images of HEK-293T cells after rAAV vector infection.

### Animal Surgery for Virus Injection

Mice were anesthetized with sodium pentobarbitone (100 mg/kg) and individually placed in the stereotaxic apparatus (Stoelting Co., Ltd.). Two microliters of rAAV-nNOS or rAAV-GFP was stereotaxically injected (0.3 μl/min) bilaterally into the BLA (anterior/posterior: 1.5 mm, medial/lateral: ± 3.5 mm, and dorsal/ventral: 4.0 mm) using a glass microsyringe. To prevent virus backflow, the pipette was left in the brain for 10 min after completion of the injection.

Seven days after injection of rAAV vectors, mice were randomly selected from each experimental group and analyzed for immunofluorescence, quantitative real-time polymerase chain reaction (qRT-PCR), and Western blot analysis to evaluate the efficiency of rAAV vectors interventions. The expression of nNOS was also quantified at 21 days after injection.

### Immunofluorescence

Immunohistochemical protocols were used in this study, as we have been previously described (Wang et al., 2017). Mice were anesthetized and perfused transcardially with 4% paraformaldehyde. Brains were removed from the skull and post-fixed overnight at 4°C. Coronal sections (40 μm) were cut on a vibratome (Leica Microsystems, Mannheim, Germany) in 0.1-M phosphate-buffered saline (PBS). Sections incubated in a blocking solution that consisted of 2% bovine serum albumin, 0.25% Triton X-100, and 5% goat serum in 0.1-M PBS. After that, sections were incubated with a mouse anti-GFP (Invitrogen, Carlsbad, CA, United States) in a dilution of 1:2,000 at 4°C overnight. The Alexa Fluor 488-conjugated donkey anti-goat immunoglobulin G (Invitrogen, Carlsbad, CA, United States) in 1:600 dilutions was used as secondary antibodies. After three washes with 1 × PBS, sections were mounted on glass slides with 75% glycerol in PBS and coverslipped. Sections were scanned with the Zeiss 900 laser scanning microscope (Carl Zeiss, Jena, Germany).

### RNA Extraction and Quantitative Real-Time Polymerase Chain Reaction

Laser microdissection of BLA was performed using a Laser PALM-Zeiss Microbeam system (PALM, Oberkochen, Germany) configured on an inverted Axio Observer Microscope. The expression of nNOS in the BLA tissues from rAAV-nNOS-treated mice and the respective controls was analyzed using qRT-PCR. Total RNA was extracted using TRIZOL Reagent (Invitrogen, Carlsbad, CA, United States) and reverse transcribed using the SuperScript III First-Strand Synthesis System (Invitrogen). Complementary DNA was used for qRT-PCR, which was performed using the SYBR^®^ GREEN PCR Master Mix (Kapa Biosystems, Woburn, MA, United States). nNOS expression was used as a control for messenger RNA (mRNA) expression. Gene expression changes were quantified using the 2^–ΔΔ*CT*^ method (Wang et al., 2018a). The primer sequences were as follows: nNOS forward 5′-ACC CAA CGT CAT TTC TGTCC-3′ and reverse 5′-AAGGTGGTCTCCAGGTGTGT-3′; glyceraldehyde 3-phosphate dehydrogenase forward 5′-TGTTGCCATCAATGACCCCTT-3′ and reverse 5′-CTCCACGACGTACTCAGCG-3′.

### Immunoblot Analysis

Laser-assisted microdissection of BLA was carried out, as mentioned earlier. BLA tissues were homogenized in radioimmunoprecipitation assay buffer [50 mM Tris-HCl (pH 7.5), 150 mM NaCl, 1% NP40, 1% sodium deoxycholate, 0.1% sodium dodecyl sulfate, and 1-mM phenylmethylsulfonyl fluoride]. Homogenates were centrifuged at 10,000g at 4°C for 15 min. Forty micrograms of soluble fractions was loaded and electrophoresed on an 8% Tris-HCl gel under reducing conditions. Proteins were then transferred onto polyvinylidene difluoride membranes (Millipore, Milford, MA, United States). The membranes were probed with the primary antibodies overnight at 4°C: nNOS (1:5,000; Sigma) and glyceraldehyde 3-phosphate dehydrogenase (1:1000; CST, Beijing, China). The membranes were incubated with horseradish peroxidase-conjugated goat anti-rabbit/mouse IgG (1:5000; ZSGB-Bio, Beijing, China) for 2 h at room temperature. Membranes were developed using the ECL Detection Kit (Thermo Fisher Scientific, Rockford, IL, United States). The blot intensities were analyzed using Quantity One software (Bio-Rad, Hercules, CA, United States).

### Behavioral Assessments

Three weeks after intra-BLA injections of rAAV vectors, the social interaction, marble burying, self-grooming, and open-field tests in mice (23–25 g) were carried out for four consecutive days. At 24 h after completion of the open-field task, the mice received either electrophysiological recordings or were sacrificed for Western blot analysis. All behavioral recordings were performed using camera-assisted ANY-Maze software (Stoelting Co., Wood Dale, IL, United States).

### Sociability and Social Novelty

The tests for sociability and social novelty preference were carried out in a three-chamber apparatus, as previously described with minor modification (Moy et al., 2004). All mice were habituated to the test chamber for 20 min and 1 day before the behavioral test. The three-chamber apparatus was a black plastic box (45 × 28 × 23 cm). Before the sociability test, the mouse was free to explore the apparatus for 8 min. After the habituation phase, an unfamiliar (stranger 1) mouse was placed in the plastic cylinder. An identical empty cylinder was placed in the right chamber. The cylinders (11 cm in diameter and 15 cm in height) were transparent, and their walls contained holes that allowed the animal to sniff each other. The subject mouse was placed in the central chamber and allowed to explore the entire chambers for 10 min freely.

In the social novelty preference test, a second novel mouse, stranger 2, was placed in the right chamber, which was empty in the previous session. The subject mouse was allowed to explore the apparatus for 10 min freely. The time spent in each chamber and the time spent sniffing or interacting with the stranger mouse were recorded. Additionally, the numbers of entries into all chambers were analyzed.

### Marble-Burying

The marble-burying was measured according to published protocols (Wang et al., 2018b). Subject mice were acclimated to a clean cage (30 × 18 × 16 cm) filled with ∼6 cm thickness of wood-chips over a 20 min period. Afterward, 15 clean glass marbles were placed into the cage equidistant from each other. Mice were then left with the marbles for 15 min, and after this period, marbles were counted as buried if > 67% of the marble was covered with bedding.

### Self-Grooming

Self-grooming testing was performed for repetitive and compulsive behavior, which is another core symptom of ASD (Kalueff et al., 2016). The conditioning chamber is made up of a rectangular box (44 × 22 × 10 cm) with 2 cm of bedding. The time mice spent grooming was calculated for 10 min. Grooming behavior was considered as face-wiping, scratching/rubbing of head and ears, and full-body grooming.

### Open Field

The open-field test was performed, as we previously described (Wang et al., 2018b). Mice were placed individually in the center of the open field to explore freely for a 5-min session. Mice were placed in the corner of the open-field apparatus (40 × 40 × 32 cm) facing the apparatus wall. The time spent in the center area (15 × 15 cm) and total distances traveled were calculated for 10 min.

### Acute Slice Preparation and Electrophysiology

Three weeks after viral infusion, mice were decapitated under deep ether anesthesia, and electrophysiological analysis was performed on mouse brain slices as described earlier (Selimbeyoglu et al., 2017). The brain was removed and immediately placed in an ice-cold artificial cerebrospinal fluid (ACSF) that consisted of (in millimoles) 75 sucrose, 85 NaCl, 2.5 KCl, 1.25 NaH_2_PO_4_, 4 MgCl_2_, 24 NaHCO_3_, 25 glucose, and 0.5 CaCl_2_ and equilibrated with 95% O_2_/5% CO_2_. Horizontal slices 300 μm thick were made for recordings from nNOS-containing interneurons using Leica VT1200 S vibrating microtome (Wetzlar, Germany). Slices from the BLA were sorted and incubated in a chamber filled with oxygenated ACSF at 34°C for 40 min. Slices then transferred to room temperature to an oxygenated standard ACSF solution containing 123 mM NaCl, 26 mM NaHCO_3_, 3 mM KCl, 1 mM MgCl_2_, 2 mM CaCl_2_, 1.25 mM NaH_2_PO_4_, and 11 mM glucose.

GFP-positive cells in the BLA were visualized with an Olympus BX51WI system microscope equipped with an infrared optical system, a CCD camera, and a 60× water-immersion objective (Zeiss, Axioskop2 FS Plus). Tips were filled with a potassium gluconate-based internal solution that consisted of [in millimoles: 140 K-gluconate, 8 NaCl, 0.2 CaCl_2_, 2 EGTA, 2 Mg-ATP, 0.5 Na_3_-GTP, and 10 HEPES (pH 7.4 adjusted with KOH, 290 mOsm)]. The pipette resistance was 3–5 MΩ with the intracellular solution. sEPSC was voltage-clamped at −70 mV in the presence of 20 μM bicuculline. For assessments of mEPSC, ACSF contained 0.5 mM tetrodotoxin (TTX, Sigma) and 10 μM bicuculline. Data were sampled at 10 kHz filtered at 3 kHz using an Axopatch 700B amplifier equipped with a 1440A Digidata (Molecular Devices, Sunnyvale, CA, United States). The series resistance was less than 12 MΩ, and cells were excluded if the series resistance changed by more than 20% during the recording. Data were collected 5 min after the currents were stably maintained. The electrophysiology recordings were analyzed using Clampfit 10.3 software (Molecular Devices).

### Biochemical Measurement of Surface Expression on the Ionotropic Glutamate Receptors

We used fluorescence-activated cell sorting to select GFP-positive interneurons (Cormack et al., 1996). Membrane proteins of GFP-expressing cells were prepared with a Mem-PER Plus Membrane Protein Extraction Kit (Thermo Scientific, Waltham, MA, United States) according to the manufacturer’s instructions. Briefly, membrane proteins of nNOS-positive interneurons were homogenized in the permeabilization buffer using a Dounce tissue grinder and incubated at 4°C for 15 min. Total cell lysates were centrifuged at 10,000 × g for 20 min at 4°C, and supernatant fractions were collected. To measure total expression, 20-μg proteins in the supernatant were removed. For surface expression, 160 μg proteins in the supernatant were incubated with 100 μl of 50% NeutrAvidin agarose (Pierce Chemical Company) overnight at 4°C. The bound proteins were resuspended in sodium dodecyl sulfate sample buffer and boiled. Quantitative Western blots were performed on both surface and total proteins of NMDA receptor subtypes (GluN2A and GluN2B), AMPA receptors (GLuA1 and GLuA2/3), and PSD-95.

### Statistical Analysis

All data are presented as the means ± standard error. We performed statistical analysis using GraphPad 7 (GraphPad Software, La Jolla, California, United States). In the sociability and social novelty preference tests, the statistical significance was analyzed by two-way analysis of variance followed by Tukey’s *post hoc* test for multiple comparisons. Otherwise, comparisons between the two groups were carried out using Student’s two-tailed paired or unpaired *t*-tests. *p* < 0.05 was considered statistically significant.

## Results

### Confirmation of Neuronal Nitric Oxide Synthase Knockdown

**FIGURE 1:**
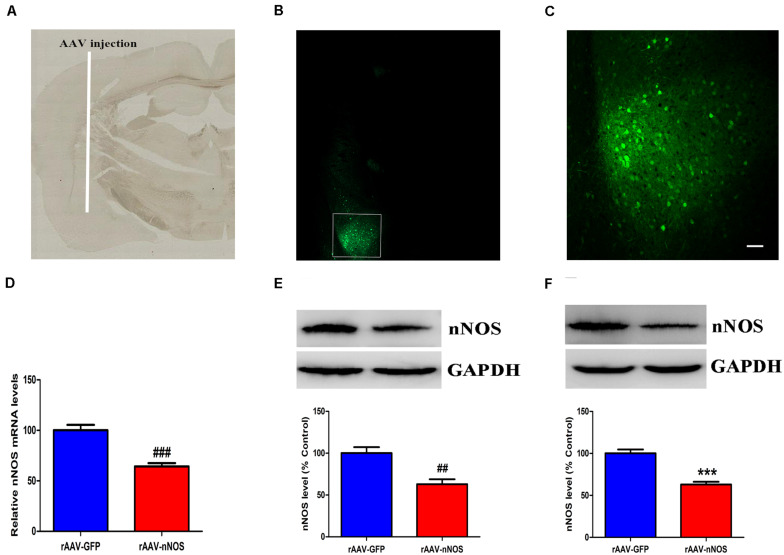
Distribution of green fluorescent protein (GFP) and the impact of rAAV-nNOS on endogenous nNOS expression in mice. **(A)** BLA injection site at low magnification. **(B)** GFP signals observed in BLA interneurons at 7 days post-injection confirmed successful nNOS vector expression. **(C)** Higher-magnification view of boxed BLA area in **(B)**. Scale bar, 60 μm. Mice with rAAV-nNOS injection showed significantly lower expression of nNOS mRNA **(D)** and protein **(E)** compared with the control group. **(F)** Three weeks after the injection of rAAV vectors, the expression of nNOS protein in the rAAV-nNOS group was significantly lower than controls. ^###^*p* < 0.01, ^##^*p* < 0.01, ***p < 0.001, compared with rAAV-GFP-treated group. *n* = 5. Student’s *t*-tests were performed. All data are presented as means ± SEM.

We first assessed whether the nNOS shRNA (rAAV-nNOS) vectors were successfully administered in the BLA. Mice treated with scramble virus (rAAV-GFP) infusions at the corresponding time points were used as controls. On the seventh day after the injection, GFP expression of the virus was confirmed by examining tissue slices that contained BLA transduced with the rAAV-nNOS-GFP virus using fluorescence microscopy (Figures 1A–C). In contrast to control group, mice infected with rAAV-nNOS showed significantly lower expression of nNOS mRNA (*p* < 0.001, Figure 1D) and lower expression of nNOS protein (*p* < 0.01, Figure 1E). These findings indicated the injection of rAAV-nNOS into the mouse BLA effectively intervened with the endogenous expression of nNOS. Further, 3 weeks after the injection of lentiviral vectors, Western blot analyses demonstrated that the nNOS protein levels in the rAAV-nNOS group were significantly lower than those in the rAAV-GFP group (*p* < 0.001, Figure 1F). Collectively, these findings strongly highlight the stability of the lentiviral vectors. Notably, the number of nNOS interneurons of rAAV-GFP-treated mice was (24 ± 2.1) cells/mm^2^. However, in the BLA of mice infected with rAAV-nNOS, the number of nNOS-containing cells was (17 ± 2.5) cells/mm^2^ (*p* < 0.01). nNOS activity was also decreased in nNOS-deficient mice compared with that in the corresponding controls (*p* < 0.01, Supplementary Figure 4).

### Neuronal Nitric Oxide Synthase Deficiency Induces Social Deficits in Mice

**FIGURE 2:**
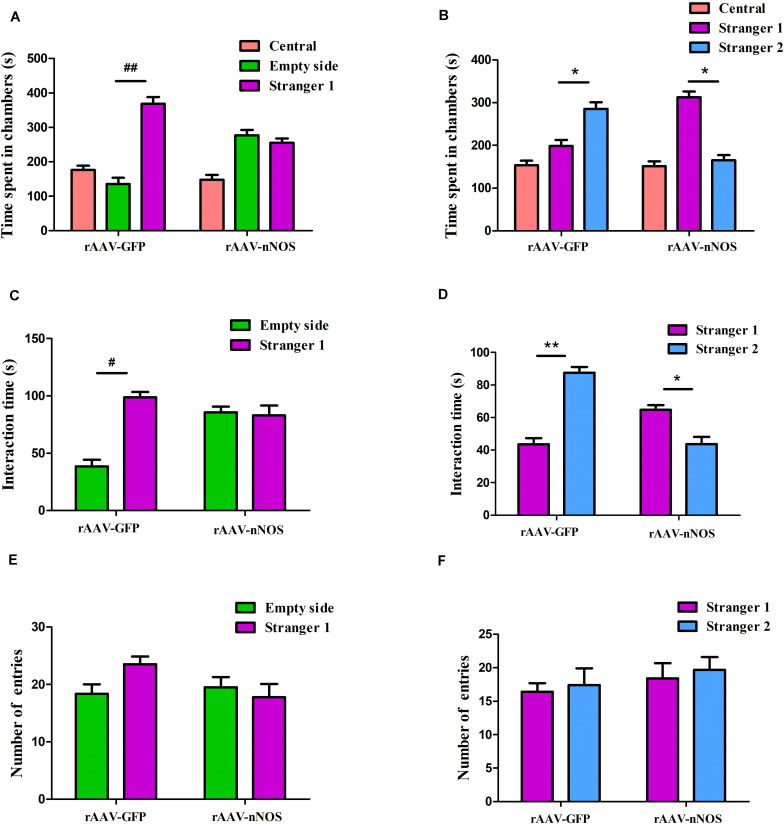
nNOS-deficient mice exhibit social impairment in the three-chamber test. **(A)** Time spent in each of the three chambers was analyzed. **(B)** Active interaction times with the empty cylinder and a stranger 1 in the session were also evaluated. rAAV-GFP-injected mice spent more time in the compartment with stranger 1 than in the empty cylinder and displayed more interaction with the mouse than with the empty cylinder, indicating normal sociability. **(C)** nNOS-deficient mice spent equal durations of both total time and interaction time in the chamber with the empty cylinder and stranger 1. **(D)** In the preference for social novelty test, the time spent in each compartment was analyzed. rAAV-GFP-treated mice spent more time in the chamber with stranger 2 than in the compartment with stranger 1. However, mice injected with rAAV-nNOS spent less time with stranger 2 than in the chamber with stranger 1. **(E)** Duration of interaction time was measured with stranger 1 and the novel 2 mouse. rAAV-GFP-treated mice showed a marked increase in the duration of interaction with stranger 2 as compared with stranger 1. Nonetheless, nNOS-deficient mice displayed a preference for stranger 1. **(F)** No difference was observed in the number of entries into compartments with stranger 1 and stranger 2 in the rAAV-nNOS group compared with the rAAV-GFP group. ^##^*p* < 0.01, ^#^*p* < 0.05, compared with empty cylinder; **p < 0.01, *p < 0.05, as compared with the chamber containing stranger 1. *n* = 10. Statistical significance was analyzed by two-way ANOVA followed by Tukey’s *post hoc* test. All data are presented as means ± SEM.

To explore behavioral changes of nNOS knockdown in native mice, we characterized the animals in the sociability and social novelty preference tests. Impairments in social interaction are the most recognizable manifestation of autistic behaviors in humans (Won et al., 2012). In the sociability test session, we found that mice infected with rAAV-GFP spent a notably longer time in the chamber with an unfamiliar animal than in the empty chamber (*p* < 0.01, Figure 2A). However, nNOS-deficient mice did not spend a longer time in the chamber with the stranger 1 mouse (*p* > 0.05). Further, we found that rAAV-GFP-treated mice spent markedly more time in close interaction with the stranger 1 mouse, indicating normal social ability (*p* < 0.05, Figure 2B). rAAV-nNOS-treated mice showed no marked preference between these two cylinders, indicating that they did not exhibit interest in the unfamiliar mouse (*p* > 0.05). No difference was found in the number of entries into these two chambers in the rAAV-nNOS group in comparison with that in the rAAV-GFP group (*p* > 0.05, Figure 2C).

In the test for social novelty preference, we found that rAAV-GFP-injected mice showed a preference for exploring the compartment with the stranger 2 as compared with the chamber containing stranger 1 (*p* < 0.05, Figure 2D). Similarly, rAAV-GFP-treated mice spent more time in social interactions with the stranger 2 compared with controls (*p* < 0.05, Figure 2E). nNOS -deficient mice spent a longer time in the chamber and social interactions with the first unfamiliar mouse than the second novel mouse (*p* < 0.01, *p* < 0.05). In agreement with the decreased social interaction in the sociability test, it was found that the social novelty preference was affected in nNOS-deficient mice. However, both rAAV-GFP-injected mice and rAAV-nNOS-injected mice displayed a comparable number of entries into the compartment with stranger 1 and stranger 2 (*p* > 0.05, Figure 2F).

### Neuronal Nitric Oxide Synthase Deficient Mice Exhibit Deficits in Stereotyped/Repetitive and Anxiety-Like Behaviors

**FIGURE 3:**
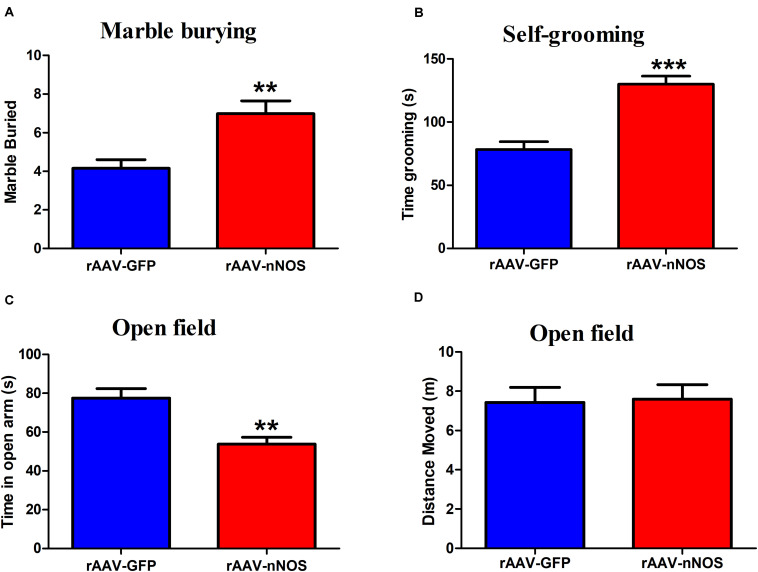
rAAV-nNOS induces stereotyped/repetitive and anxiety-like behaviors in native mice. **(A)** In the marble burying test, a deficiency of nNOS increased the number of marbles buried compared with that in rAAV-GFP-treated mice. **(B)** In the grooming test, mice with rAAV-nNOS injection prolonged time spent grooming compared with corresponding controls. **(C,D)** In the open-field test, nNOS knockdown increased the time spent in the center as compared with that in control mice. *n* = 12. **p < 0.01, ***p < 0.001, compared with rAAV-GFP-treated group. Student’s *t*-test assessed statistical significance. Data are shown as means ± SEM.

The marble-burying and self-grooming tests were adopted to study repetitive and compulsive-like behaviors (Angoa Pérez et al., 2013; Bey et al., 2018). We found that the injection of rAAV-nNOS resulted in an increased total number of marbles buried in mice when compared with controls (*p* < 0.01, Figure 3A). nNOS deficiency also substantially increased the time spent grooming compared with the corresponding controls (*p* < 0.001, Figure 3B).

To further determine whether mice injected with rAAV-nNOS displayed anxiety-related behavior, we performed an open-field test. As illustrated in Figures 3C,D, reduced nNOS expression apparently decreased the time spent in the center of open field compared with the rAAV-GFP group (*p* < 0.01). No significant difference was detected in the distance moved between two groups (*p* > 0.05). Altogether, the discussed results illustrate that deficiency of nNOS induces core behaviors relevant to ASD in mice, such as social disturbance, enhanced repetitive behaviors, and anxiety.

### Downregulation of Neuronal Nitric Oxide Synthase Inhibits Excitatory Synaptic Transmission

**FIGURE 4:**
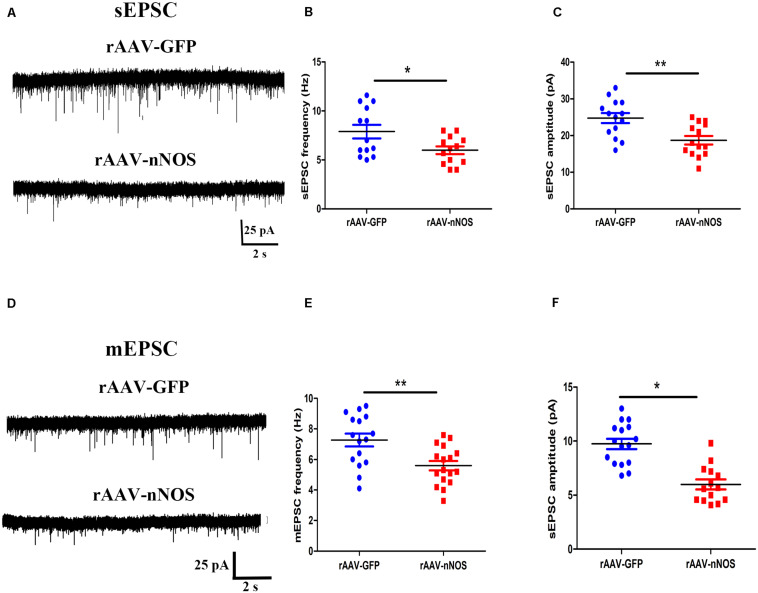
Effects of nNOS downregulation on excitatory synaptic transmission in slice from nNOS interneurons in mice. **(A)** Continuous recordings of spontaneous excitatory postsynaptic current (sEPSC) from typical cells in mice injected with rAAV-GFP (upper) and rAAV-nNOS (bottom). Summary graph of mEPSC frequency **(B)** and amplitude **(C)** for cells from control and rAAV-nNOS-injected mice. rAAV-GFP: *n* = 14 cells in 5 mice; rAAV-nNOS: *n* = 14 cells in five mice. **(D)** Representative recordings of miniature excitatory postsynaptic current (mEPSC) in control (upper) and rAAV-nNOS-treated mice (bottom). Summary graph of mEPSC frequency **(E)** and amplitude **(F)** in rAAV-GFP-treated and nNOS-deficient mice. *n* = 16 cells in 5 mice; rAAV-nNOS: *n* = 17 cells in 5 mice. *p < 0.05, **p < 0.01, compared with rAAV-GFP-injected animals, unpaired *t*-test. Data are presented as means ± SEM.

The altered glutamatergic synaptic transmission has been postulated to underlie the autistic features (Lee et al., 2017), prompting us to test whether the nNOS knockdown affects excitatory postsynaptic currents (sEPSC and mEPSC) in nNOS-expressing interneurons of BLA slices from control and rAAV-nNOS-injected mice. We made successful whole-cell recordings from nNOS-expressing interneurons in rAAV-nNOS-injected mice and control slices. As illustrated in Figures 4A–C, the mEPSC frequency and amplitude were robustly decreased in the rAAV-nNOS-treated mice as compared with those in the controls (*p* < 0.05, *p* < 0.01).

Further, we found the significantly lower mean frequency and amplitude of mEPSC in nNOS-positive interneurons from the rAAV-nNOS-injected mice compared with those in the rAAV-GFP group (*p* < 0.01, *p* < 0.05, Figures 4D–F). Thus, our observations support the idea that nNOS deficiency markedly depresses glutamatergic synaptic transmission in nNOS interneurons.

### Decreased Receptor Proteins on the Excitatory Postsynaptic Membrane in Neuronal Nitric Oxide Synthase-Deficient Mice

**FIGURE 5:**
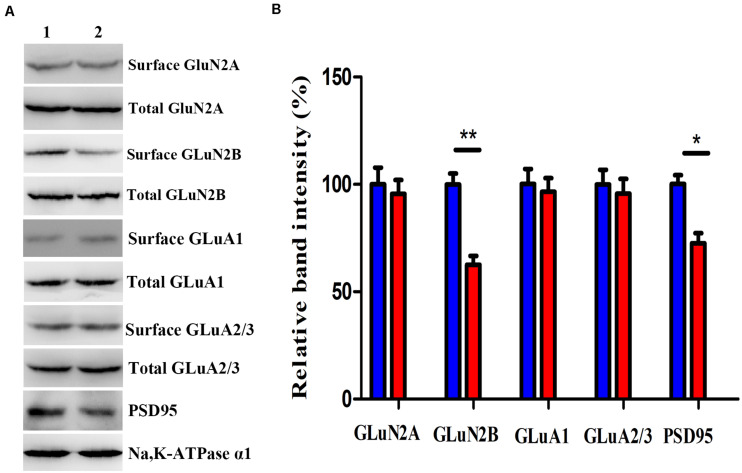
Effects of nNOS deficiency on GluN2A, GluN2B, GluA1, GluA2/3, and PSD-95 levels in native mice. **(A,B)** Expression of GLuN2B membrane protein and PSD-95 in the rAAV-nNOS group was lower than that in control mice. Surface expression of GLuN2A, GluA1, and GluA2/3 protein was significantly unaltered between the rAAV-nNOS group and controls. Band 1, rAAV-GFP-treated group; Band 2, rAAV-nNOS-exposed group. *n* = 6, **p < 0.01, *p < 0.05, compared with rAAV-GFP-injected controls, Student’s *t*-test. All data are shown as mean ± SEM.

We investigated the GluN2A, GluN2B, GluA1, GluA2/3, and PSD-95 levels, which predominantly mediate excitatory synaptic transmission in the BLA, in mice after virus intervention. Western blot analysis demonstrated that the nNOS deficiency imposed a significant decrease in the levels of GluN2B membrane protein and PSD-95 in nNOS interneurons compared with those in the rAAV-GFP group (*p* < 0.01, *p* < 0.05), whereas the total GluN2B level remained comparable with that of control mice (*p* > 0.05, Figures 5A,B). We found no apparent difference in the surface expression of GluN2A, GluA1, and GluA2/3 in nNOS interneurons between the two groups (*p* > 0.05). Hence, these results indicate that nNOS knockdown might decrease the expression of GluN2B surface protein and PSD-95, which could further influence excitatory synaptic transmission.

## Discussion

In the present study, we demonstrate that nNOS deficiency induces several signs of ASD-associated phenotypes in mice, as evidenced by aberrant social interactions, augmentation of stereotypes, and anxiety. Importantly, nNOS-deficient mice also exhibit reduced glutamatergic synaptic transmission. This is concurrent with lower surface levels of GLuN2B and PSD-95 in the nNOS-containing interneurons in rAAV-nNOS-treated animals. These abnormal changes in the BLA may contribute to ASD-relevant behavioral dysfunction.

A growing body of literature has identified a range of ASD-like behavioral abnormalities in animals exposed to VPA *in utero* (Kotajima Murakami et al., 2019). This includes the previous studies from our lab, where we identified impaired social interaction, enhanced repetitive behavior, and anxiety of mice models relative to ASD (Wang et al., 2018b). Further, we have shown reduced nNOS interneuron numbers and nNOS expression in the BLA from VPA-treated mice (Wang et al., 2018a). In the current study, we hypothesized that knockdown of nNOS might contribute to the aberrational features observed in native animals. To test our hypothesis, we used a viral-mediated approach to downregulate nNOS in the BLA. Our results revealed for the first time that nNOS knockdown mice exhibited ASD-related symptoms, including reduced social interaction, enhanced stereotypes, and anxiety-like behavior, strongly reminiscent of behavioral phenotypes of nNOS knockout mice (Gao and Heldt, 2015). Consistent with this, decreases in parvalbumin (PV) interneuron numbers and PV mRNA levels have been reported in autistic human and animal brains. PV knockout mice also demonstrate the core symptoms of ASD (Wöhr et al., 2015). AT-rich interactive domain 1B (Arid1b) haploinsufficiency mice are known to display a reduced number of cortical GABAergic interneurons and exhibit ASD-associated behaviors, including defects in social and anxiety behavior (Jung et al., 2017). Taken together, these results indicate that the decrease in endogenous nNOS levels might be sufficient to induce ASD-related symptoms. However, further researches are required to more specifically target whether nNOS deficiency contributes to the dysfunction of synaptic transmission and behavioral characteristics in VPA-treated mice.

Intriguingly, we found that nNOS deficiency resulted in decreased excitatory synaptic transmission in nNOS interneurons in mice. Our data were congruent with studies showing glutamatergic synaptic defects in the mouse hippocampus after maternal immune activation (Ito et al., 2010). Slices in visual cortex pyramidal neurons were shown to display strong suppression of mEPSC amplitude and frequency from the Ube3a (2 × Tg) transgenic mouse model of ASD (Smith et al., 2011; Platzer et al., 2017). Deneault et al. (2018) reported that there was a reduced sEPSC frequency in ASD-susceptibility genes (ATRX- and ASTN2)-null neurons. In agreement with these observations, we found that knockdown of nNOS led to decreased excitatory synaptic inputs in animals, indicating a relatively reduced capacity in BLA information transfer.

Given the findings mentioned earlier, we investigated the GLuN2B expression caused by nNOS knockdown in mice. GLuN2B is the primarily expressed subunits of NMDA in the interneurons that mediate excitatory neurotransmission in the amygdala (Nörenberg et al., 2012; Bacq et al., 2018). Of particular noteworthy, the present data showed that the expression of GLuN2B membrane protein was found to be downregulated in nNOS-deficient interneurons in animals. It is worth mentioning that nNOS exerted no appreciable effect on the total GLuN2B protein levels. Consistent with our results, accumulating evidence reported that pathogenic *de novo* variant in the gene encoding NMDA receptor subunits GLuN2B has been identified in autistic subjects (Liu et al., 2017; Platzer et al., 2017). Remarkably, a recent study observed that the GluN2B variant (S1413L) led to impairments in NMDA receptor trafficking and reduced mEPSC amplitude, which may contribute to abnormal phenotypes in individuals with ASD (Liu et al., 2017). Moreover, mice heterozygous for T-box brain 1 (Tbr1), encoding a transcription factor with targets the GluN2B subunit, display NMDA receptor hypofunction and social deficits responsive to clioquinol (Lee et al., 2015b, 2017). As such, we propose that a decrease in surface expression of GLuN2B-containing NMDA receptor after reducing endogenous nNOS expression is responsible for reduced glutamatergic synaptic transmission, which may be involved in behavioral aberrations associated with ASD.

We found a significant reduction of PSD-95 protein levels in nNOS-deficient mice. Consistent with this notion, the expression of GluN2B and PSD-95 was decreased in the prefrontal cortex of Shank3-deficient non-human primate (Zhao et al., 2017). nNOS and PSD-95 are coexpressed in the BLA (Yi et al., 2017; Zhou et al., 2018). Tsai et al. demonstrated that ASD-linked genes mediate synapse elimination via a synaptic scaffold PSD-95 (Tsai et al., 2012). We speculate that nNOS may regulate the surface NMDA receptor fraction through its interaction with PSD-95. Notably, compelling evidence suggests that protein phosphatase 1-mediated dephosphorylation of the GluN2B through interaction with PDZ of PSD-95 decreases GluN2B synaptic content in an activity-dependent manner (Chiu et al., 2019). Additional research is needed to determine whether nNOS-PSD-95 interaction reduces GluN2B surface protein by promoting dephosphorylation of the GluN2B interacting with PDZ in nNOS-deficient mice.

## Conclusion

To summarize, we demonstrate that the virus-mediated knockdown of nNOS contributes to the core ASD-associated deficits ranging from the excitatory synaptic transmission to behavioral features that are associated with multiple symptomatic domains. This provides novel and interesting information for the ASD research community and can be further investigated as a role for nNOS as a possible therapeutic intervention through genetic or pharmacological manipulations.

## Data Availability Statement

The datasets generated for this study are available on request to the corresponding author.

## Ethics Statement

The animal study was reviewed and approved by the Institutional Animal Ethics Committee of Shandong University, following the NIH Guidelines for the Care and Use of Laboratory Animals.

## Author Contributions

XW and YS designed the research and wrote the manuscript. CG and QF performed the viral infusion surgeries. LG and DM conducted the behavioral characterization and analyzed the data. LeL and LiL carried out qRT-PCR and western blotting. JX performed electrophysiological experiments and analyzed the data as well. YZ, JL, and HZ critically revised the manuscript. All authors have read and approved the final version of the manuscript.

## Conflict of Interest

The authors declare that the research was conducted in the absence of any commercial or financial relationships that could be construed as a potential conflict of interest.
