# Supplementary material for: Neuronal Nitric Oxide Synthase Knockdown Within Basolateral Amygdala Induces Autistic-Related Phenotypes and Decreases Excitatory Synaptic Transmission in Mice
Source: Front Neurosci. 2020 Aug 31;14:886. doi: 10.3389/fnins.2020.00886 (PMC7488195; doi:10.3389/fnins.2020.00886)
Supplement: Supplementary file 1 [file Data_Sheet_1.pdf]

Supplementary Materials

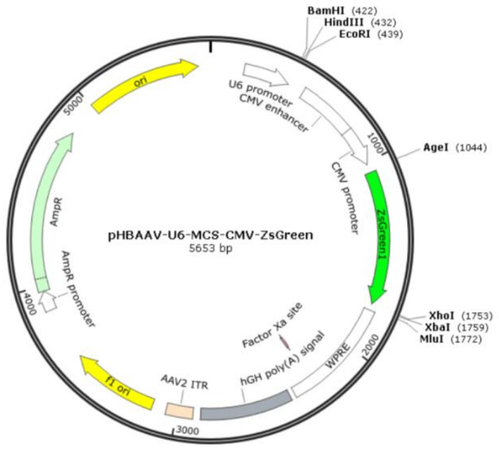

Figure S1 | Spectrum of a graph of AAV vector.

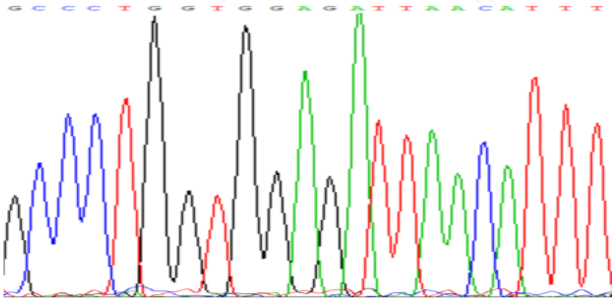

Figure S2 | Identification of recombinant AAV vector by DNA sequencing.

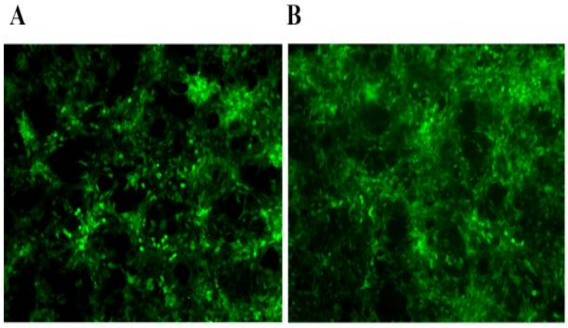

Figure S3 | Fluorescence images of HEK293 cells following rAAV vector infection (×100). (A) uninfected nNOS cells. (B) RNAi-nNOS (infection with nNOS-GFP-rAAV).
