# Supplementary material for: Neuronal Nitric Oxide Synthase Knockdown Within Basolateral Amygdala Induces Autistic-Related Phenotypes and Decreases Excitatory Synaptic Transmission in Mice
Source: Front Neurosci. 2020 Aug 31;14:886. doi: 10.3389/fnins.2020.00886 (PMC7488195; doi:10.3389/fnins.2020.00886)
Supplement: Supplementary file 2 [file Data_Sheet_2.pdf]

Supplementary Materials

MATERIALS AND METHODS

nNOS activity assay

For nNOS activity, BLA samples were homogenized and centrifuged at 12,000 g for 15 min at 4 ℃. The supernatant was ultracentrifuged at 12,000 g for 20 min and the protein concentration was quantified by a BCA protein assay kit (Pierce, Rockford, IL, USA). Total nNOS activity in the filtrates was measured using a nNOS assay kit (Jiancheng Bioengineering Co., Nanjing, China). nNOS activity was expressed as unit (U).

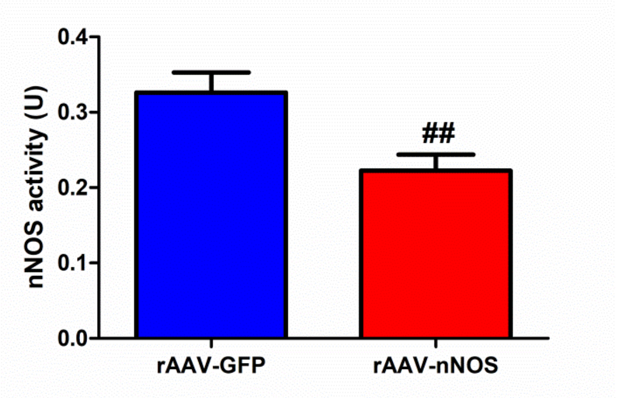

Figure S4 | Effect of nNOS down-regulation on nNOS activity in native mice. Three weeks after the injection of rAAV vectors, nNOS activity in rAAV-nNOS group was lower compared to controls. $^{\#\#}p < 0.01$, compared to rAAV-GFP-treated group. $n = 6$. Student's t-tests were performed. Data are shown as means ± SEM.
